# An update on the biological and pharmacological activities of diosgenin

**DOI:** 10.17179/excli2017-894

**Published:** 2018-01-02

**Authors:** Jae Kwang Kim, Sang Un Park

**Affiliations:** 1 Division of Life Sciences and Convergence Research Center for Insect Vectors, Incheon National University, Incheon 22012, Korea; 2 Department of Crop Science, Chungnam National University, 99 Daehak-ro, Yuseong-gu, Daejeon, 34134, Korea

## ⁯

Dear Editor,

Diosgenin, a phytosteroid saponin, is found at high levels in several plant species, including *Costus* *speciosus*, *Smilax menispermoidea*, *Trigonella foenum*, species of *Paris*, *Aletris*, *Trigonella*, and *Trillium*, and many species of *Dioscorea *(Patel et al., 2013[25]; Chen et al., 2011[4]).

Fujii and Matsukawa first discovered diosgenin within *Dioscorea tokoro *Makino in 1935 (Djerassi et al., 1952[7]). The biosynthesis of steroidal saponins such as diosgenin in plants has not yet been reported in detail, although cholesterol was found to be a precursor of this compound. Cholesterol is formed from lanosterol and some of the reactions involved are catalyzed by cytochrome P450 systems. Vaidya et al. (2013[29]) suggested that diosgenin might be formed from squalene-2,3-oxide in two ways: from lanosterol via cholesterol, and from cycloartenol via the formation of sitosterol (Ciura et al., 2017[6]).

In the pharmaceutical industry, diosgenin is the principal precursor compound in the manufacture of several synthetic steroidal drugs (Chen et al., 2015[5]). It also represents a promising bioactive biomolecule that exhibits various biological properties; these include hypolipidemic, hypoglycemic, antioxidant, anti-inflammatory, and antiproliferative activities (Jesus et al., 2016[14]). Diosgenin has therefore attracted considerable attention in recent years within the pharmaceutical, functional food, and cosmetic industries. Here, we summarize recent studies performed to evaluate the biological and pharmacological activities of diosgenin (Table 1(Tab. 1); References in Table 1: Badalzadeh et al., 2015[1]; Bhuvanalakshmi et al., 2017[2]; Chen et al., 2016[3]; Fang et al., 2015[8]; Folwarczna et al., 2016[9]; Hao et al., 2015[10]; Haratake A et al., 2017[11]; Hua et al., 2016[12]; Huang et al., 2017[13]; Jiang et al., 2016[15]; Junchao et al., 2017[16]; Kim et al., 2016[17]; Liu et al., 2016[19]; Liu et al., 2017[18]; Lv et al., 2015[20]; Masood-Ur-Rahman et al., 2017[21]; Mischitelli et al., 2016[22]; Naidu et al., 2015[23]; Nie et al., 2016[24]; Pi et al., 2017[26]; Selim and Al Jaouni, 2015[27]; Tikhonova et al., 2015[28]; Wang et al., 2015[30]; Wang et al., 2017[31]; Xie et al., 2015[32]; Zhang et al., 2016[33]; Zhao et al., 2016[34]; Zheng et al., 2016[35]; Zhou et al., 2017[36]). 

## Acknowledgements

This work was supported by Korea Institute of Planning and Evaluation for Technology in Food, Agriculture, Forestry and Fisheries(IPET) through Advanced Production Technology Development Program, funded by Ministry of Agriculture, Food and Rural Affairs (MAFRA) (116115-03-1-CG000). This research was supported by the Bio & Medical Technology Development Program of the National Research Foundation (NRF) funded by the Ministry of Science, ICT & Future Planning (2016M3A9A5919548).

## Conflict of interest

The authors declare no conflict of interest.

## Figures and Tables

**Table 1:**
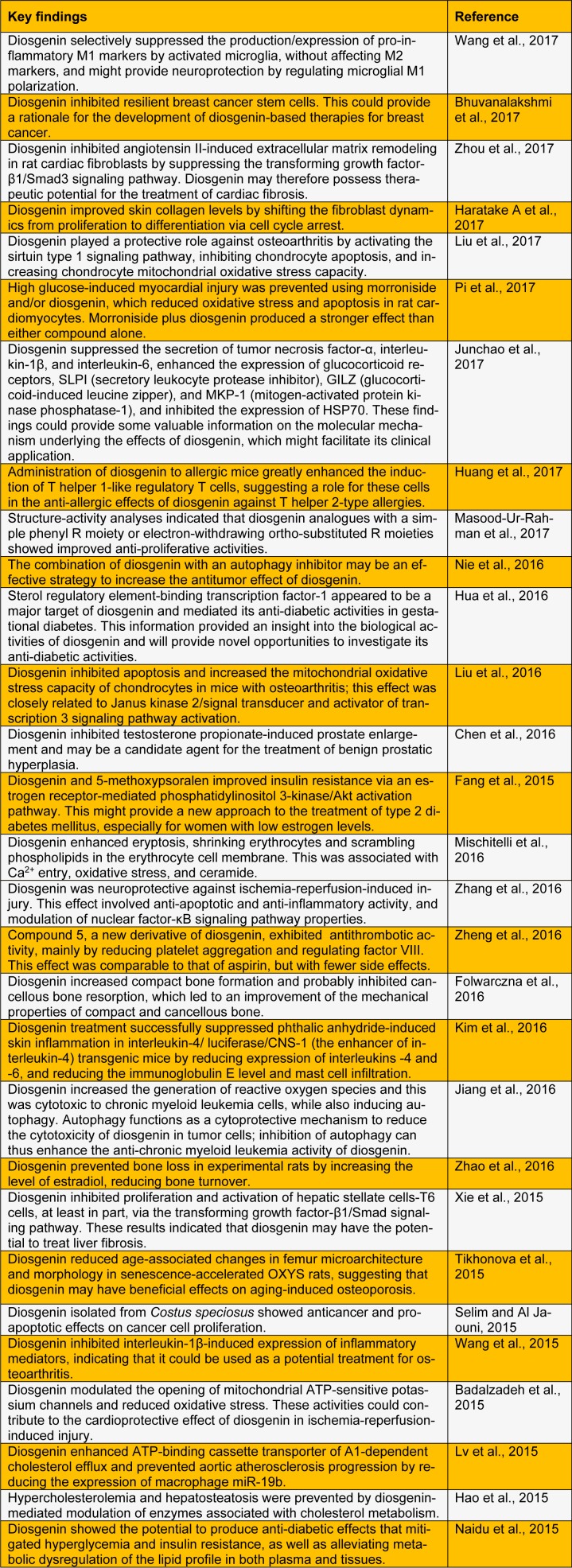
Recent studies of the biological and pharmacological activities of diosgenin
